# Analysis of Brachypodium miRNA targets: evidence for diverse control during stress and conservation in bioenergy crops

**DOI:** 10.1186/s12864-018-4911-7

**Published:** 2018-07-20

**Authors:** Karl R. Franke, Skye A. Schmidt, Sunhee Park, Dong-Hoon Jeong, Monica Accerbi, Pamela J. Green

**Affiliations:** 1 Department of Biology and Delaware Biotechnology Institute, University of Delaware, 15 Innovation Way, Newark, DE 19711 USA; 2 Department of Plant and Soil Sciences and Delaware Biotechnology Institute, University of Delaware, 15 Innovation Way, Newark, DE 19711 USA; 3 Department of Life Science, Hallym University, Chuncheon, Republic of Korea

**Keywords:** PARE, Degradome, SmRNA, miRNA, Abiotic stress, Brachypodium, Sorghum, Switchgrass, Bioenergy

## Abstract

**Background:**

Since the proposal of *Brachypodium distachyon* as a model for the grasses, over 500 Bdi-miRNAs have been annotated in miRBase making Brachypodium second in number only to rice. Other monocots, such as switchgrass, are completely absent from the miRBase database. While a significant number of miRNAs have been identified which are highly conserved across plants, little research has been done with respect to the conservation of miRNA targets. Plant responses to abiotic stresses are regulated by diverse pathways many of which involve miRNAs; however, it can be difficult to identify miRNA guided gene regulation when the miRNA is not the primary regulator of the target mRNA.

**Results:**

To investigate miRNA target conservation and stress response involvement, a set of PARE (Parallel Analysis of RNA Ends) libraries totaling over two billion reads was constructed and sequenced from Brachypodium, switchgrass, and sorghum representing the first report of RNA degradome data from the latter two species. Analysis of this data provided not only PARE evidence for miRNA guided cleavage of over 7000 predicted target mRNAs in Brachypodium, but also evidence for miRNA guided cleavage of over 1000 homologous transcripts in sorghum and switchgrass. A pipeline was constructed to compare RNA-seq and PARE data made from Brachypodium plants exposed to various abiotic stress conditions. This resulted in the identification of 44 miRNA targets which exhibit stress regulated cleavage. Time course experiments were performed to reveal the relationship between miR393ab, miR169a, miR394ab, and their respective targets throughout the first 36 h of the cold stress response in Brachypodium.

**Conclusions:**

Knowledge gained from this study provides considerable insight into the RNA degradomes and the breadth of miRNA target conservation among these three species. Additionally, associations of a number of miRNAs and target mRNAs with the stress responses have been revealed which could aid in the development of stress tolerant transgenic crops.

**Electronic supplementary material:**

The online version of this article (10.1186/s12864-018-4911-7) contains supplementary material, which is available to authorized users.

## Background

Environmental stresses such as drought, high salinity, cold, and heat have a negative impact on today’s crop yields by up to 70% [1]. Studies done by the Food and Agriculture Organization in 2007 found that only 3.5% of land area was free from environmental constraint [2]. Continued reduction in arable land, water resources, and increased global warming lead to a prediction of further yield reduction in the future [3]. These issues are further compounded in bioenergy crops such as sorghum (*Sorghum bicolor)* and switchgrass (*Panicum virgatum)* as a new source of energy. The success of traditional breeding approaches to improve stress tolerance has not been adequate and a move to transgenic approaches is necessary [1]. Having an understanding of how plants sense stress in the environment, activate the appropriate signaling networks, and subsequently make the molecular changes necessary to adapt is critical to the development of cultivars that can survive these harsher conditions.

Unfortunately, many bioenergy crops can be very difficult to work with in a laboratory setting due to their large size, growth requirements, and extended generation times. Both sorghum and switchgrass can grow to over 2.5 m in height and have generation times of over 12 weeks [4]. Additionally, they lack many of the traits which allow for functional genomic studies such as having a small diploid genome with limited repetitive DNA. Switchgrass is a complex polyploid with a genome of over 1300 Mb. While sorghum is diploid, its genome is also large, at just under 700 Mb. Arabidopsis has served as an excellent plant model system for many years, but as a dicot, it is not representative of monocot plants. As a monocot, rice (*Oryza sativa*) has also been promoted as a model for temperate grasses and a significant amount of research has made available many tools and resources. However, while rice is a much closer relative than Arabidopsis, this tropical cereal does not have many of the biological traits common to the temperate crops such as perenniality, injury tolerance, freezing tolerance, and pathogen resistance [5].

Due to these and other issues, Draper et al. proposed Brachypodium (*Brachypodium distachyon)* be used as a model system in 2001 [5]. The arguments made for Brachypodium’s use were quite compelling. Much like Arabidopsis, Brachypodium is very easy to grow in a laboratory setting with minimal requirements. Plants are self-fertile and have a short generation time of 8–12 weeks [4]. The genome of Brachypodium is also much simpler than many other grasses in that not only is it small in size at around 270 Mb, but is also diploid. As a member of the Pooideae subfamily, Brachypodium offers a model system that is not only easy to work with like Arabidopsis, but is also more closely related to our species of interest than rice.

In our previous study of Brachypodium, we used 17 small RNA libraries to identify 116 microRNAs (miRNAs), many of which are conserved among other plants as well as some unique to Brachypodium [6]. MiRNAs are small ~ 21 nt RNA molecules which interact with Argonaute (AGO) proteins to form RNA-induced silencing complexes (RISCs) [7, 8]. The RISCs direct the post-transcriptional gene regulation of a target mRNA by means of complementary base pairing between the target and the miRNA. In plants, this regulation takes place predominantly via a site-specific endoribonucleolytic cleavage of the target mRNA between the 10th and 11th nucleotides relative to the 5′ end of the miRNA; however, translational repression is also possible. This site-specific cleavage allowed us to use Parallel Analysis of RNA ends (PARE) [9] to capture and sequence the 3′ decay intermediates from these events and provide experimental evidence for the miRNA-mediated cleavage of 264 predicted Brachypodium miRNA target sites [6]. MiRNAs have been shown to be involved in many aspects of plant development [10, 11]. With roles involving the formation root, stem, leaf, and floral organs, control of cell division, regulation of hormone responses, patterning, and even regulation of miRNA biogenesis, it is not surprising that miRNAs have been found to regulate stress responses as well [12–17].

A number of miRNAs have been shown to play a role in the stress responses in various plant systems, including Brachypodium. A study done in 2009 showed induction of miR397, miR169e, and miR172 during cold stress, but at that point no analysis was done on the effects of the target mRNAs [18]. The same group published another study in 2013 demonstrating changes under cold conditions in the abundances of some miRNAs as well as changes in mRNA target levels and decay intermediates for miR393 targeting a *TIR1-like* mRNA and miR396 targeting a number of uncharacterized genes; however, miR169e and miR397 were not shown to be cold responsive in this second study [19]. The association of miR393 with cold stress in Brachypodium is not surprising as it has been shown to be induced in other stresses such as drought in Arabidopsis and rice. Additionally, its target, *TIR1*, aids in regulation by auxin, a hormone known to play a role in cold stress [20]. Drought stress responsive miRNAs in Brachypodium have also been previously identified: miR896 was shown to be induced under drought and decreased levels of a predicted target involved in alcohol metabolism were also observed [21], and miR169j was shown to be downregulated under drought conditions [22], an association also seen in Arabidopsis [23].

To have a more complete understanding of the stress responses of temperate grasses, it is most useful to know not only which miRNAs are induced or repressed, but also the response of the target mRNA. When both are known, the effect of the miRNA on the stress response is more clear, but the regulation of miRNA guided cleavages are most often validated in instances where the miRNA is the primary regulator of its target mRNA. This type of regulatory mechanism is easier to validate as it results in significant changes to target mRNA abundance; however, miRNAs commonly function to incrementally modulate the expression of their targets [7]. These relationships can be more difficult to identify since the overall abundances of the miRNAs and/or targets may only change slightly despite changes in miRNA guided cleavage. Overcoming this difficulty could reveal many potential targets for the creation of transgenic crops with higher stress tolerance.

In this study, we revealed a remarkably high level of miRNA target conservation by providing evidence for cleavage of over a thousand conserved target mRNAs across Brachypodium, sorghum, and switchgrass. Going even further, PARE libraries made from Brachypodium exposed to various abiotic stress conditions allowed for the identification of 44 unique mRNAs exhibiting changes in miRNA guided cleavage during the stress responses. We gained a deeper understanding of the regulatory mechanisms of two of these miRNA/mRNA interactions by characterizing the abundance of the RNAs throughout a cold stress time course. Finally, we show PARE evidence that these mechanisms are conserved in switchgrass and sorghum.

## Results

### Sequencing of small RNA, PARE and RNA-seq libraries from Brachypodium, sorghum, and switchgrass

To gain a deeper understanding of the RNA degradomes of Brachypodium, sorghum, and switchgrass, 34 PARE libraries were constructed from two biological replicates of various tissues, as well as plants that had been exposed to various stress conditions. Illumina HiSeq technology allowed for these libraries to be sequenced to a total depth over 2 billion reads (Table 1). Adapter sequences were trimmed using custom Perl scripts and the reads were mapped to their respective genomes using Bowtie [24–28]. 1.7 billion reads were aligned successfully with zero mismatches resulting in an average of nearly 7 million distinct genome-mapped reads per library. The RNA samples from Brachypodium plants which had been exposed to abiotic stress conditions were also used to construct RNA-seq libraries to allow for a direct comparison between Brachypodium’s transcriptomes and degradomes under these conditions (Additional file 1: Table S1). Ten RNA-seq libraries were constructed and sequenced to a total depth of 151 million reads with an average of 91% of reads uniquely mapped to the genome using STAR [29]. While a number of studies have investigated the miRNAs present in switchgrass [30–33], none have been annotated in miRBase; we constructed eight switchgrass small RNA libraries to help identify any conserved miRNAs present in the genome (Table 1).

**Table 1:** Summary statistics of PARE and smRNA libraries

		Trimmed		Genome Matched		cDNA Matched	
Condition	ID#	Distinct^a^	Total^b^	Distinct^c^	Total^d^	Distinct^e^	Total^f^
Brachypodium PARE							
Root Bio#1	BDI20	2,273,863	10,551,854	1,600,582	9,263,616	1,576,585	7,451,753
Leaf Bio#1	BDI21	1,226,270	13,696,309	682,819	12,251,858	658,393	9,549,413
Stem Bio#1	BDI23	3,499,449	16,005,365	1,416,469	12,416,968	1,350,442	11,930,438
Panicle Bio#1	BDI25	4,256,035	28,940,535	3,415,326	26,535,542	3,355,513	23,614,486
Root Bio#2	BDI381	14,892,253	71,328,838	10,909,520	64,296,408	10,072,279	62,003,648
Leaf Bio#2	BDI288	15,673,931	93,529,302	8,204,778	80,615,543	7,675,634	75,965,360
Stem Bio#2	BDI289	20,330,230	106,942,289	11,417,928	93,078,760	10,527,104	85,961,342
Panicle Bio#2	BDI287	13,605,701	70,071,025	10,619,657	64,290,369	10,165,770	53,396,674
Control Bio#1	BDI283	18,739,274	116,856,674	8,033,068	99,574,648	7,475,766	94,337,424
Control Bio#2	BDI490b	11,609,931	57,136,919	9,220,303	51,975,771	8,871,646	44,558,561
Cold Bio#1	BDI507	14,094,807	71,335,970	10,300,680	63,667,922	9,476,866	60,202,779
Cold Bio#2	BDI542	5,954,379	26,243,974	4,873,635	23,735,487	4,626,612	23,513,295
Drought Bio#1	BDI284	16,895,332	110,043,868	8,551,529	94,797,698	7,833,753	70,824,614
Drought Bio#2	BDI514	6,549,245	30,377,458	4,954,837	27,533,267	4,509,215	21,352,193
Heat Bio#1	BDI491	9,064,716	36,739,900	7,008,303	32,960,526	6,557,997	24,894,799
Heat Bio#2	BDI544	12,502,104	71,856,374	10,147,454	65,621,210	9,309,732	64,252,301
Submergence Bio#1	BDI508	19,248,274	143,926,223	13,013,551	129,521,309	12,049,556	117,577,209
Submergence Bio#2	BDI493	11,085,014	47,501,130	8,186,439	42,683,558	7,802,211	38,304,004
Sorghum PARE							
Root Bio#1	SBI617	7,408,495	47,925,541	4,667,519	40,934,942	4,158,647	33,879,769
Root Bio#2	SBI620	7,471,715	37,774,801	4,059,238	30,725,474	3,799,122	13,074,749
Leaf Bio#1	SBI619	10,557,426	57,604,765	3,881,705	41,917,126	3,274,739	36,707,076
Leaf Bio#2	SBI615	6,473,759	40,090,222	4,233,698	34,957,079	3,841,772	29,383,910
Panicle Bio#1	SBI616	6,872,817	34,611,413	3,981,997	24,631,577	3,158,582	19,666,227
Panicle Bio#2	SBI618	12,386,838	49,631,535	8,261,402	41,607,791	7,172,569	36,467,170
Control for Cold Bio#1	SBI517	3,256,249	39,956,679	2,092,580	35,409,035	1,743,771	31,035,771
Control for Cold Bio#2	SBI519	3,885,061	39,822,428	2,579,391	35,514,462	2,183,104	31,347,460
Cold Bio#1	SBI518	3,197,687	47,188,888	2,294,992	44,114,986	1,957,618	30,508,940
Cold Bio#2	SBI520	3,420,185	36,488,885	2,324,259	33,101,913	1,795,310	22,262,865
Switchgrass PARE							
Leaf Bio#1	SWI352	18,119,798	66,514,735	7,302,551	29,289,132	6,684,352	27,261,224
Leaf Bio#2	SWI498	22,300,993	114,883,981	16,237,561	100,834,434	13,901,137	91,031,475
Stem Bio#1	SWI353	20,252,951	94,311,170	5,644,700	24,757,380	5,276,609	21,054,163
Stem Bio#2	SWI500	5,712,914	18,900,653	4,315,446	16,340,546	3,600,582	14,307,880
Panicle Bio#1	SWI382	23,916,903	96,013,301	15,181,789	80,968,945	13,320,327	76,420,288
Panicle Bio#2	SWI499	3,052,670	7,845,043	2,448,779	6,885,542	2,140,816	6,234,349
Control for Cold Bio#1	SWI521	10,188,795	62,673,164	7,125,183	55,462,967	6,454,159	50,607,577
Control for Cold Bio#2	SWI523	7,480,243	42,889,534	4,344,702	33,834,492	3,456,913	29,312,647
Cold Bio#1	SWI522	8,657,953	50,614,176	4,996,684	39,012,357	3,302,826	28,359,863
Cold Bio#2	SWI524	6,617,641	42,861,290	4,203,457	36,211,923	2,949,417	24,326,833
Switchgrass smRNA							
Control for Cold	SWI560	5,575,102	33,117,391	4,036,929	29,240,850	NA	NA
Cold	SWI559	10,477,573	74,342,226	7,231,085	64,406,974	NA	NA
Control for Drought	SWI551	6,099,269	34,328,102	4,490,488	30,101,667	NA	NA
Drought	SWI552	5,288,223	34,223,739	3,807,420	30,123,899	NA	NA
Re-water	SWI553	5,202,390	30,490,652	3,728,694	26,891,552	NA	NA
Leaf	SWI561	1,021,704	5,310,089	764,472	4,710,729	NA	NA
Stem	SWI534	774,658	5,081,224	575,800	4,428,676	NA	NA
Panicle	SWI562	1,798,117	3,221,179	1,472,871	2,807,413	NA	NA

^a^The number of unique sequences found in a library after trimming. ^b^The total number of sequences found in a library after trimming. ^c^The number of unique sequences that match the genome at least once. ^d^The total number of sequences that match the genome at least once. ^e^The number of unique sequences that match the sense strand of the transcriptome at least once. ^f^The total number of sequences that match the sense strand of the transcriptome at least once

### Characterization of predicted miRNA targets in Brachypodium

**Fig. 1:**
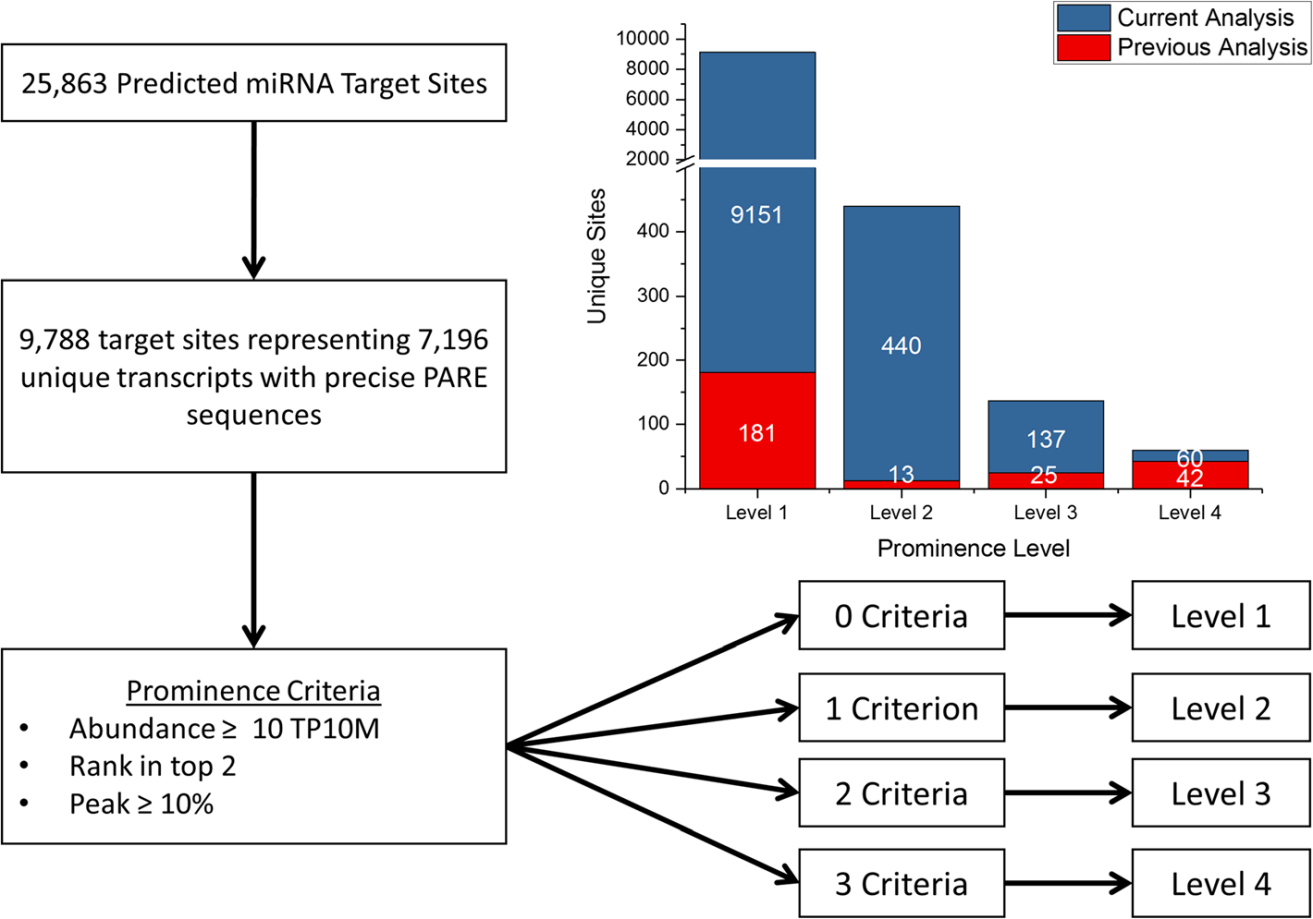
Characterization of predicted Brachypodium miRNA targets using PARE. The abundances of PARE sequences at predicted miRNA target sites were compared to the abundances of the other PARE sequences mapping the transcripts of the targets. The target sites were characterized into four distinct levels depending on how many prominence criteria were met: having an abundance greater than or equal to 10 TP10M, ranking in the top two most abundant PARE sequences mapping to the target transcript, and if the abundance of the sequence represented at least 10% of the total abundance of all PARE sequences mapping to the target transcript

While the use of Brachypodium as a model for plants such as sorghum and switchgrass has been well established since its proposal, no large scale analysis of conserved miRNA targets between these plants has been done. To this end, we sought to identify miRNA targets conserved among these species for which we could provide PARE evidence of cleavage. In our previous work, four PARE libraries provided evidence for cleavage of 264 predicted miRNA target sites [6]; however, since then a new revision of the genome has been released along with an updated annotation. With 14 additional PARE libraries, as well as an updated genome, we set out to rerun our previous analysis to discover if the number of Brachypodium miRNA targets with PARE evidence for cleavage would increase substantially with these new data. We used psRNATarget and Targetfinder [34, 35] to predict 25,863 miRNA target sites, 9788 of which had precise PARE sequences at the target site (Fig. 1). The PARE data allowed for us to characterize the sequence matching the predicted target site based on three prominence criteria: having an abundance greater than or equal to 10 TP10M (Transcripts Per 10 Million Reads), ranking in the top two most abundant PARE sequences mapping to the target transcript, and if the abundance of the sequence represented greater than 10% of the total abundance of all PARE sequences mapping to the target transcript. This analysis yielded 60 unique target sites falling into the Level 4 category, 137 in Level 3, 440 in Level 2, and 9151 in Level 1, a large increase across all categories compared to the previous analysis (Additional file 1: Table S2).

### Identification of conserved miRNA targets in switchgrass and sorghum

**Fig. 2:**
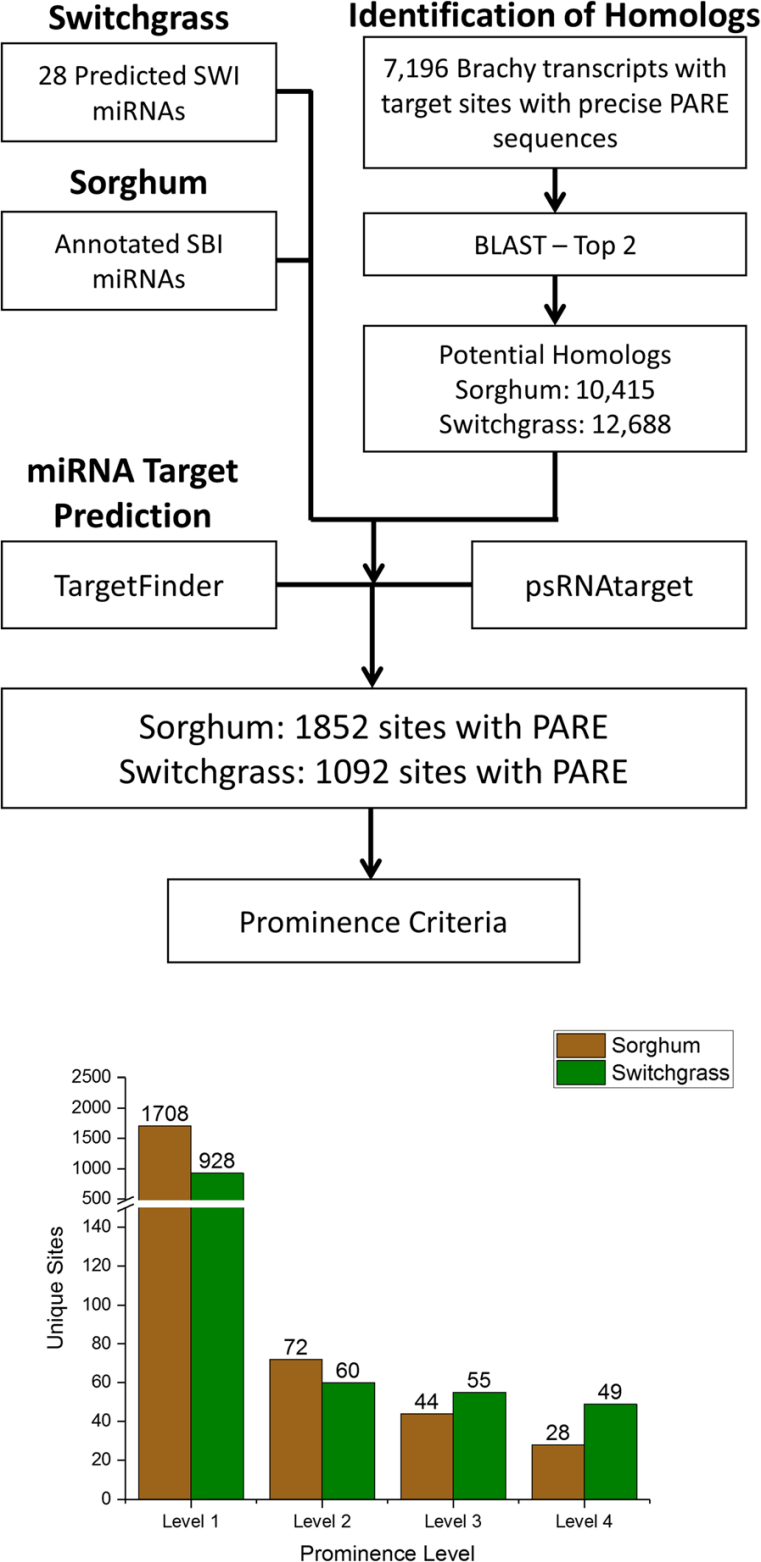
Pipeline for the identification of conserved miRNA targets. Protein sequences of Brachypodium targets with PARE evidence for miRNA guided cleavage were blasted against databases of switchgrass and sorghum proteins. The top two hits for each transcript were kept and used for miRNA target prediction along with the annotated sorghum miRNAs and 28 predicted Switchgrass miRNAs. Predicted target sites were characterized using PARE data based on the same prominence criteria described in Fig. 1

**Fig. 3:**
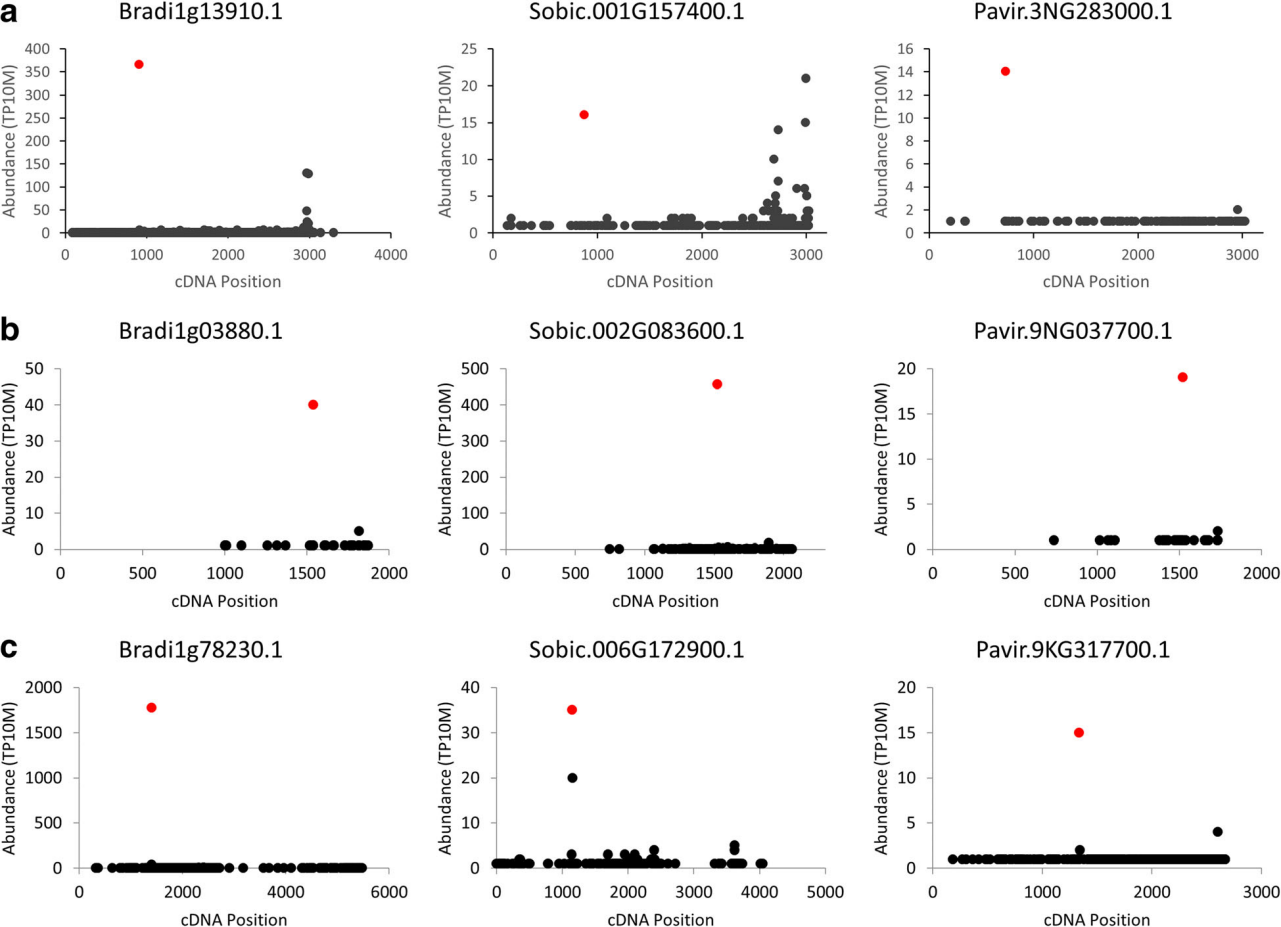
D-Plots of conserved miRNA targets. Analysis of PARE data revealed many conserved miRNA targets between Brachypodium, sorghum, and switchgrass including (**a**) miR166 guided cleavage of a HD-ZIP III transcription factor, (**b**) miR172 guided cleavage of an AP2 transcription factor, and (**c**) miR171 guided cleavage of a GRAS transcription factor. Red dots indicate the PARE sequences mapping to predicted target sites

With an updated list of Brachypodium miRNA targets we developed a pipeline, outlined in Fig. 2, to determine if any of these 7196 transcripts had homologs in sorghum or switchgrass that were also miRNA targets. Protein sequences of the Brachypodium transcripts were blasted against sorghum and switchgrass databases and the top two candidates for each transcript were identified and used as input for miRNA target prediction programs TargetFinder and psRNAtarget. While many Brachypodium and sorghum miRNAs have been annotated, miRBase21 contains no switchgrass miRNAs. We put the smRNA libraries listed in Table 1 through our Sequence Homology Pipeline for miRNA discovery [6] with updated criteria based on recent recommendation [36] to identify 28 unique conserved miRNA sequences from 84 precursors in switchgrass (Additional file 1: Table S3). In sorghum, 1852 unique target sites had PARE sequences matching the predicted target sites representing 1562 unique transcripts. In switchgrass, 1092 unique target sites had PARE evidence for cleavage within 1019 unique transcripts. After characterizing the target sites with the same criteria used in the analysis of Brachypodium targets, it became clear that not only was there a large number of miRNA targets conserved across these three species, but that the conservation was seen across all four prominence levels. Despite sorghum having a greater number of conserved miRNA target sites overall, switchgrass had a greater number of Level 3 and 4 target sites with 49 Level 4, 55 Level 3, 60 Level 2 and 928 Level 1 targets (Additional file 1: Table S4). While sorghum had 28, 44, 72, and 1708 target sites at these levels, respectively (Additional file 1: Table S5). Example D-Plots (Degradation Plots) of conserved targets can be seen in Fig. 3.

### Identification of stress regulated miRNA guided cleavage events in Brachypodium

**Fig. 4:**
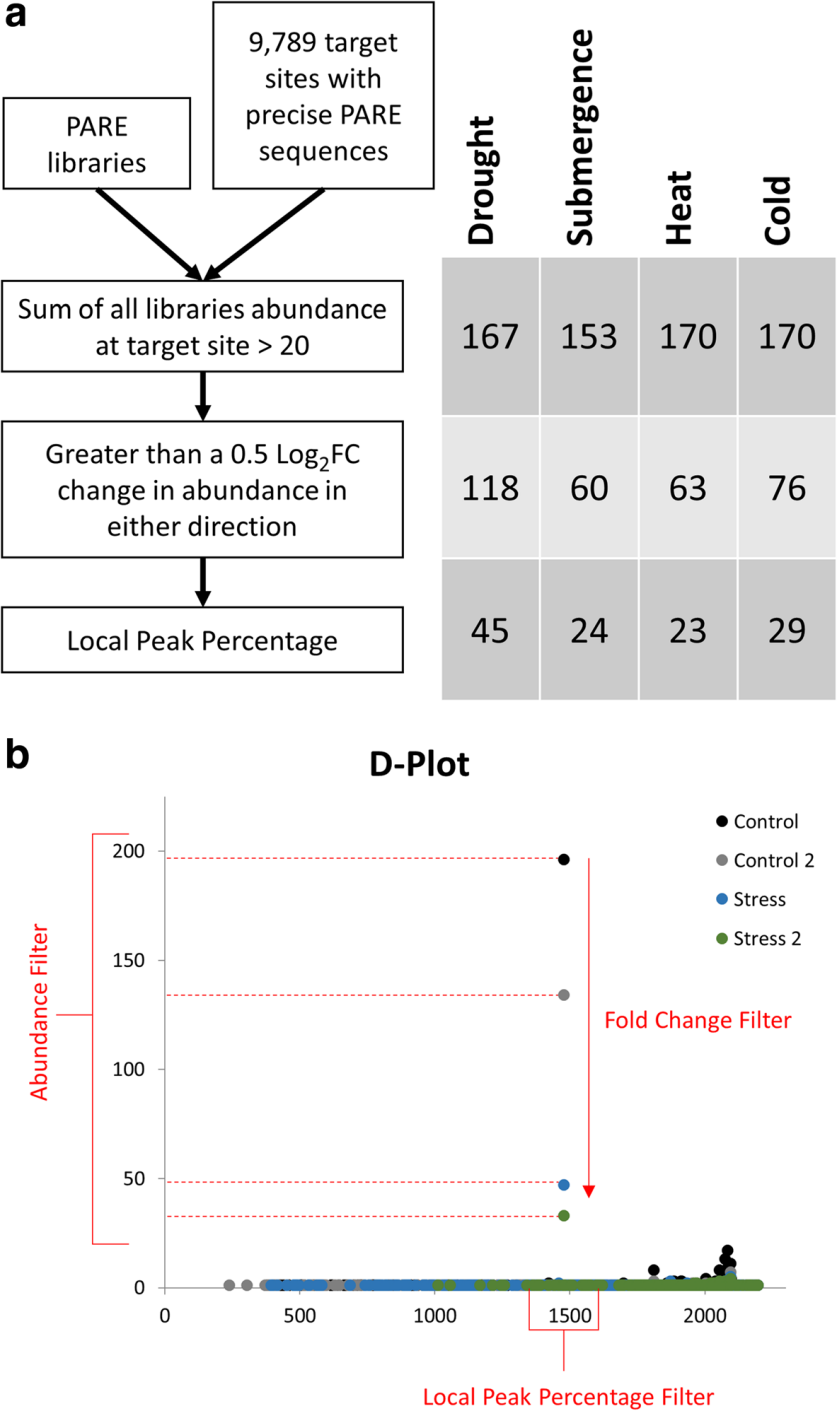
Pipeline for the identification of stress regulated miRNA guided cleavage in Brachypodium. **a** Outline of the pipeline detailing how many target sites passed each filtering step in each stress. **b** Visualization of the Abundance Filter, Fold Change Filter, and Local Peak Percentage Filter on an example D-Plot

The relatively high number of Brachypodium miRNA targets found to be conserved in sorghum and switchgrass gave us confidence that miRNA-controlled stress regulatory mechanisms observed in Brachypodium would have a high chance of being conserved in sorghum and/or switchgrass as well. To this end, we designed a pipeline to analyze Brachypodium PARE data from two biological replicates of control and each stress condition to determine which miRNA target sites showed evidence for regulation under stress (Fig. 4). Three different filters were used to identify the most promising candidates. The first two filters only considered the abundance of the specific PARE sequence mapping to the predicted target site. An abundance filter was used to ensure enough data were available to make confident determinations of regulation; potential targets with a sum of PARE abundance less than 20 TP10M across all four libraries were discarded. Next, the changes in PARE abundance between control and stress were compared; a candidate was only kept if there was at least a 0.5 log_2_ fold change in abundance in the same direction in both biological replicates. A final “Local Peak Percentage” filter compared the abundance of the PARE sequence mapping to the predicted target site to the sum of the abundances of all PARE sequences mapping within a 50 nt window around the target site. This was done to ensure the sequence at the target site was not part of the overall background decay of the target. In the condition (control or stress) which exhibited evidence for increased cleavage based on an increase in PARE abundance, the abundance of that PARE sequence needed to represent more than 25% of the total PARE abundance within the 50 nt window or the candidate was discarded. In total, this analysis identified 63 unique mRNA targets which passed all three filters: 45 were identified in drought stress conditions, 24 in submergence, 23 in heat, and 29 in cold.

### Categorization of target mRNAs based on RNA-seq

**Fig. 5:**
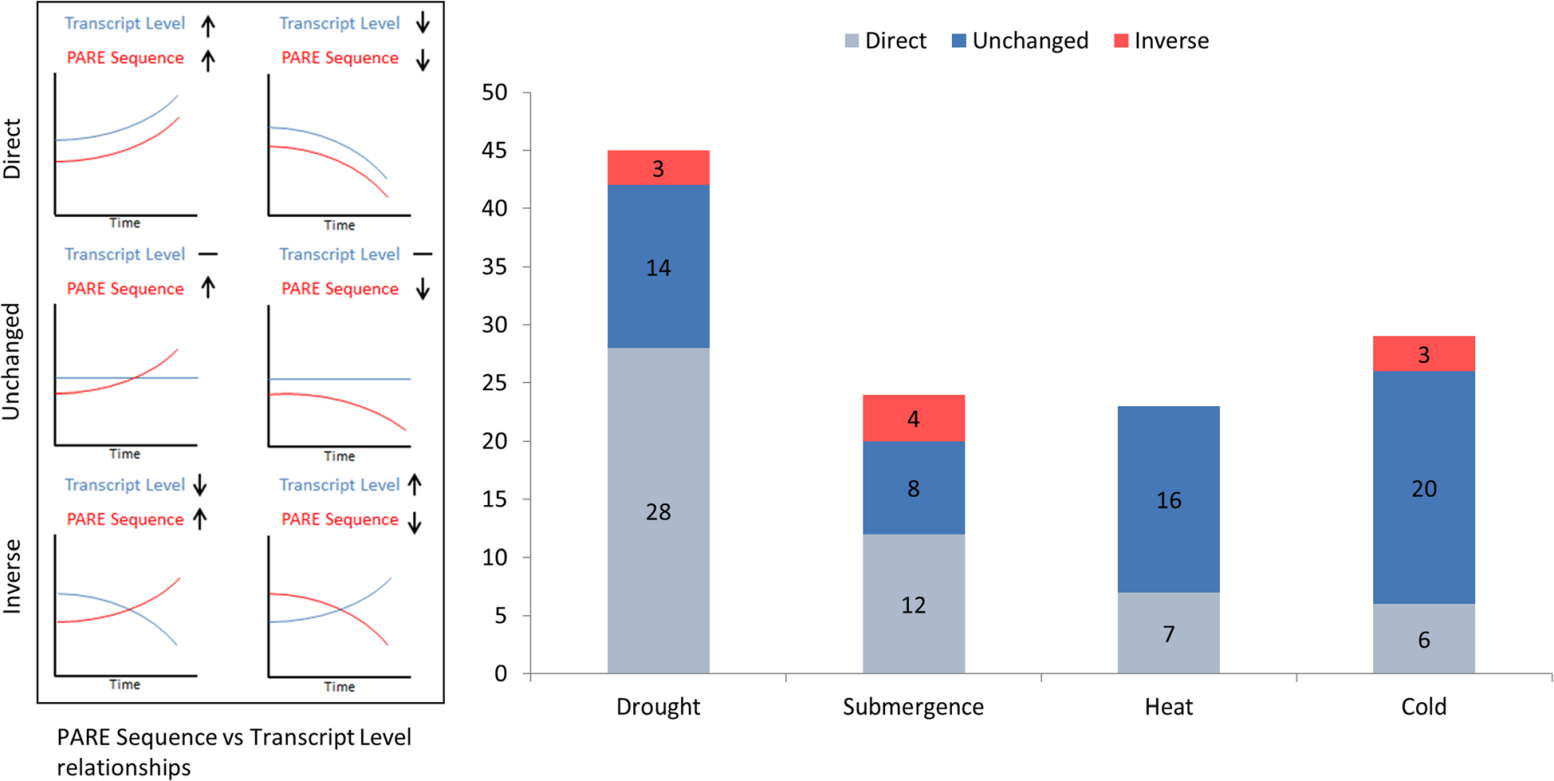
Characterization of stress associated miRNA target sites using RNAseq. For each stress condition, target sites passing all three filters of the previous pipeline were characterized based on change in PARE abundance and change in overall transcript level as observed in RNAseq. “Direct” target sites exhibited changes in PARE under stress conditions which occurred in the same direction as changes in overall transcript level. The “Unchanged” group consisted of target sites within transcripts which despite changes in PARE did not exhibit changes in transcript level. Lastly, the “Inverse” group exhibited changes in PARE under stress that occurred in the opposite direction of changes observed in RNAseq

**Fig. 6:**
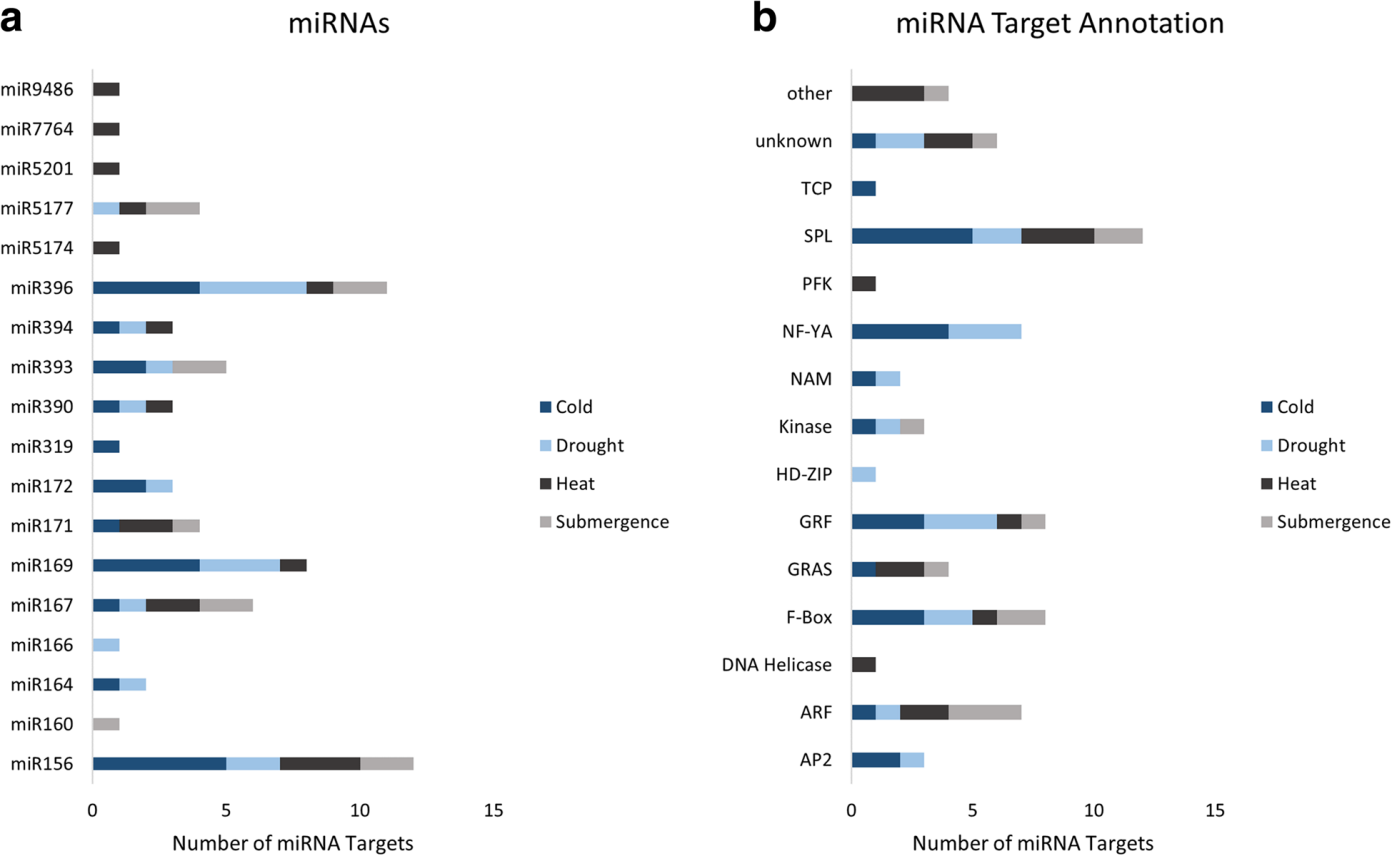
Examination of the miRNAs and target annotations in the “Unchanged” and “Inverse” groups. A large number of the miRNAs (**a**) as well as the annotations of the mRNA targets (**b**) support involvement in multiple stress responses

While our previous analysis did identify a number of miRNA targets sites with PARE sequences exhibiting changes in abundance under stress conditions, these data alone are not enough to suggest that the regulation observed is post-transcriptional. It is possible that changes in PARE abundance are due to changes in transcription that alter mRNA abundance. To determine which target mRNAs exhibited changes in PARE abundance at the target site which could not be explained by changes in transcription, we categorized the targets using RNA-seq data (Fig. 5). If the changes observed in the abundance of the PARE sequence at the predicted target site occurred in the same direction as the abundance of the mRNA transcript then the target mRNA was put in the “Direct” category. This group represented the majority of candidates in drought, 28 out of 45, as well as half of the candidates in submergence, 12 of 24. If the abundance of the target mRNA did not change between control and stress conditions, the target was put in the “Unchanged” category. Heat and cold had the majority candidates in this group with 16 and 20 respectively. Finally, if the PARE and RNA-seq data changed in opposite directions, the target was put in the “Inverse” category. Three targets from drought, four from submergence, and three from cold were categorized as “Inverse.” While the changes observed in PARE for targets in the “Direct” category could be explained by changes in transcription, that is not the case for the “Unchanged” and “Inverse” groups. Additionally, even though the abundances of the mRNA transcript levels for the “Unchanged” group were not significantly different between control and stress conditions, that does not disqualify these miRNA guided cleavage events from being involved in the stress responses. Such changes in target cleavage might be used to maintain mRNA abundances that would have been altered by other regulatory mechanisms such as changes in transcription. Many of the miRNAs targeting mRNAs in the “Unchanged” and “Inverse” groups show evidence for involvement in multiple stress responses (Fig. 6a). Targets of miR396, miR167 and miR156 appear to be involved in all four stress responses, while those of miR5177, miR394, miR393, miR390, miR171, and miR169 show evidence for involvement in three. A large amount of overlap is also seen in the annotation of the targets with various types of transcription factors representing the majority (Fig. 6b and Additional file 1: Table S6). Transcripts in the SPL, GRF, F-Box, and ARF families are represented in the miRNA targets of all four stresses. The ten candidates in the “Inverse” group can be found in Table 2.

**Table 2:** Inverse miRNA Targets

Transcript	Annotation	miRNA	Cleavage Site	psRNAT^a^ EXP	UPE	TF^b^ Score	C1^c^ Abun	E1^d^ Abun	C2^e^ Abun	E2^f^ Abun	PARE LogFC1	PARE LogFC2	RNAseq LogFC	RNAseq PValue	Stress
Bradi1g11800.5	NF-YA	miR169a-5p	1447	4.5	16.524	4	95	53	82	34	−0.842	−1.270	0.822	2.15E-04	Cold
Bradi2g35720.1	TIR1	miR393ab	1787	3	17.544	1	52	21	52	7	−1.308	−2.893	0.484	2.76E-02	Cold
Bradi2g59200.1	LCR	miR394	1479	0	20.387	0	196	47	134	33	−2.060	−2.022	0.463	3.19E-02	Cold
Bradi2g28153.1	Unknown	miR5177	1267	5	20.308	none	15	2	3	2	−2.907	−0.585	0.838	2.97E-04	Drought
Bradi2g38552.2	Unknown	miR390a-5p	767	4	7.202	none	36	11	42	13	−1.710	− 1.692	0.693	3.59E-02	Drought
Bradi3g52547.1	GRF	miR396ab	592	1	21.583	1	1085	303	1407	443	−1.840	−1.667	0.983	1.87E-04	Drought
Bradi2g35720.1	TIR1	miR393ab	1787	3	17.544	1	52	30	52	26	−0.794	−1.000	1.180	2.55E-07	Submergence
Bradi3g05510.1	SPL11	miR156a	857	2	20.607	2	107	56	58	29	−0.934	−1.000	0.559	2.18E-02	Submergence
Bradi3g50930.3	GRAS	miR171a	1475	none	none	0	51	86	43	70	0.754	0.703	−0.750	9.11E-04	Submergence
Bradi5g08680.1	F-box	miR393ab	2183	3	23.264	1	15	5	20	9	−1.585	−1.152	0.451	4.81E-02	Submergence

^a^psRNATarget Expectation. ^b^TargetFinder Score. The normalized abundance of PARE read mapping to the predicted target site in biological replicate #1 under control^c^ and stress^d^ conditions and biological replicate #2 under control^e^ and stress^f^ conditions

### Cold stress time course

**Fig. 7:**
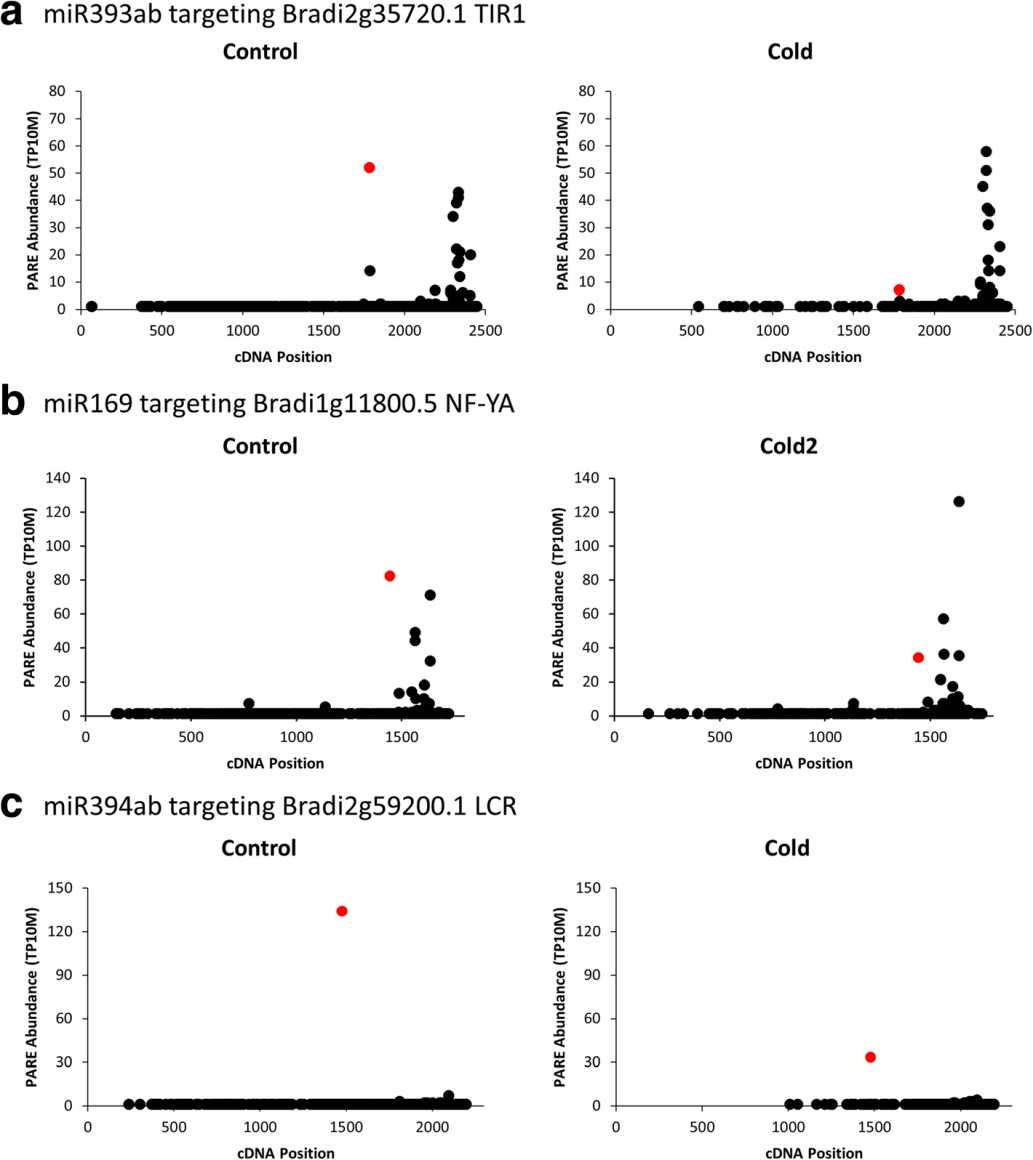
D-Plots of the cold regulated inverse group miRNA targets. PARE data showing evidence for cold regulation of the miRNA guided cleavages of (**a**) Bradi2g35720.1, (**b**) Bradi1g11800.5, and (**c**) Bradi2g59200.1. The second biological replicate can be found in Additional file 2: Figure S1. Red dots indicate the PARE sequences mapping to predicted target sites

**Fig. 8:**
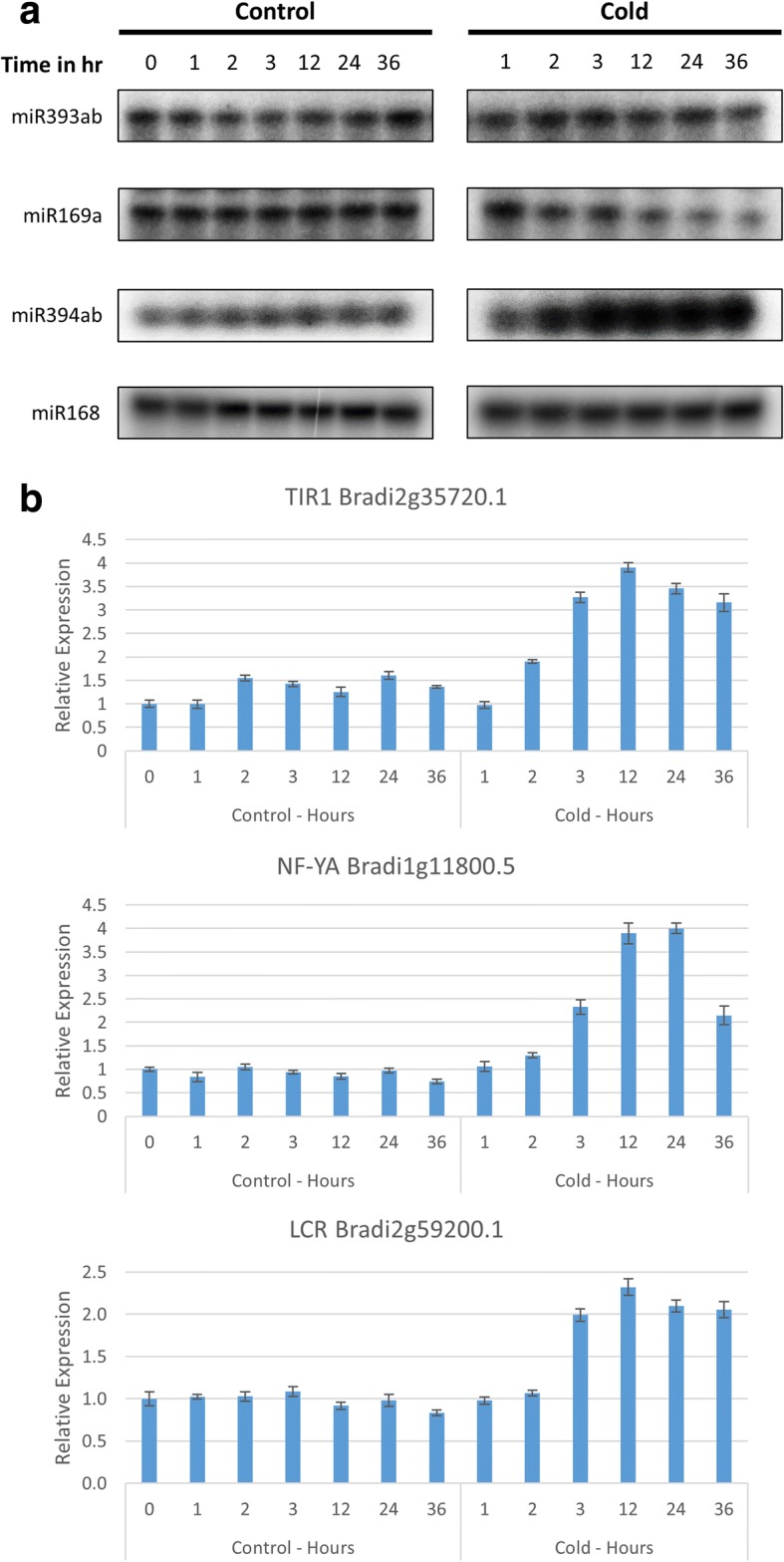
Characterization of cold regulated miRNAs and mRNA targets. **a** The abundances of miR394ab, miR169a, and miR393ab were characterized using splint ligation mediated miRNA detection throughout the cold stress time course. miR168 serves as a non-regulated control. **b** The abundances of target transcripts were characterized using qRT-PCR. Data from the second biological replicate can be found in Additional file 2: Figure S2

Cold was a prominent association among miRNAs and targets (Figs. 5 & 6). Moreover, miRNAs identified by our pipeline under cold conditions included one which had previously been implicated in the cold stress response (miR393ab) as well as two with novel responses to cold in Brachypodium (miR394 and miR169a). For these reasons we expanded our single time point analysis of cold stress to include a 36 h time course with two biological replicates for this stress. We chose to focus on the miRNAs and targets in the “Inverse” group as the miRNAs in this group have the highest chance of being the primary regulators of the target mRNAs, making correlations between changes in expression of miRNA and target mRNA more likely. We characterized the abundance of the miRNA targets, *NF-YA* (Bradi1g11800.5), *TIR1* (Bradi2g35720.1), and *LCR* (Bradi2g59200.1) using qRT-PCR (Fig. 8b). The relative expression of all targets showed an increase beginning at 3 h; results were as one might expect given the decrease in miRNA guided cleavage suggested by PARE data (Fig. 7). To characterize expression levels of the targeting miRNAs, we performed splint-ligation mediated miRNA detection [37] (Fig. 8a). Despite the observed changes in *TIR1* mRNA levels, we did not find significant changes in abundance of miR393ab; in this instance, miR393ab may not be the primary regulator of the *TIR1* transcript.

**Fig. 9:**
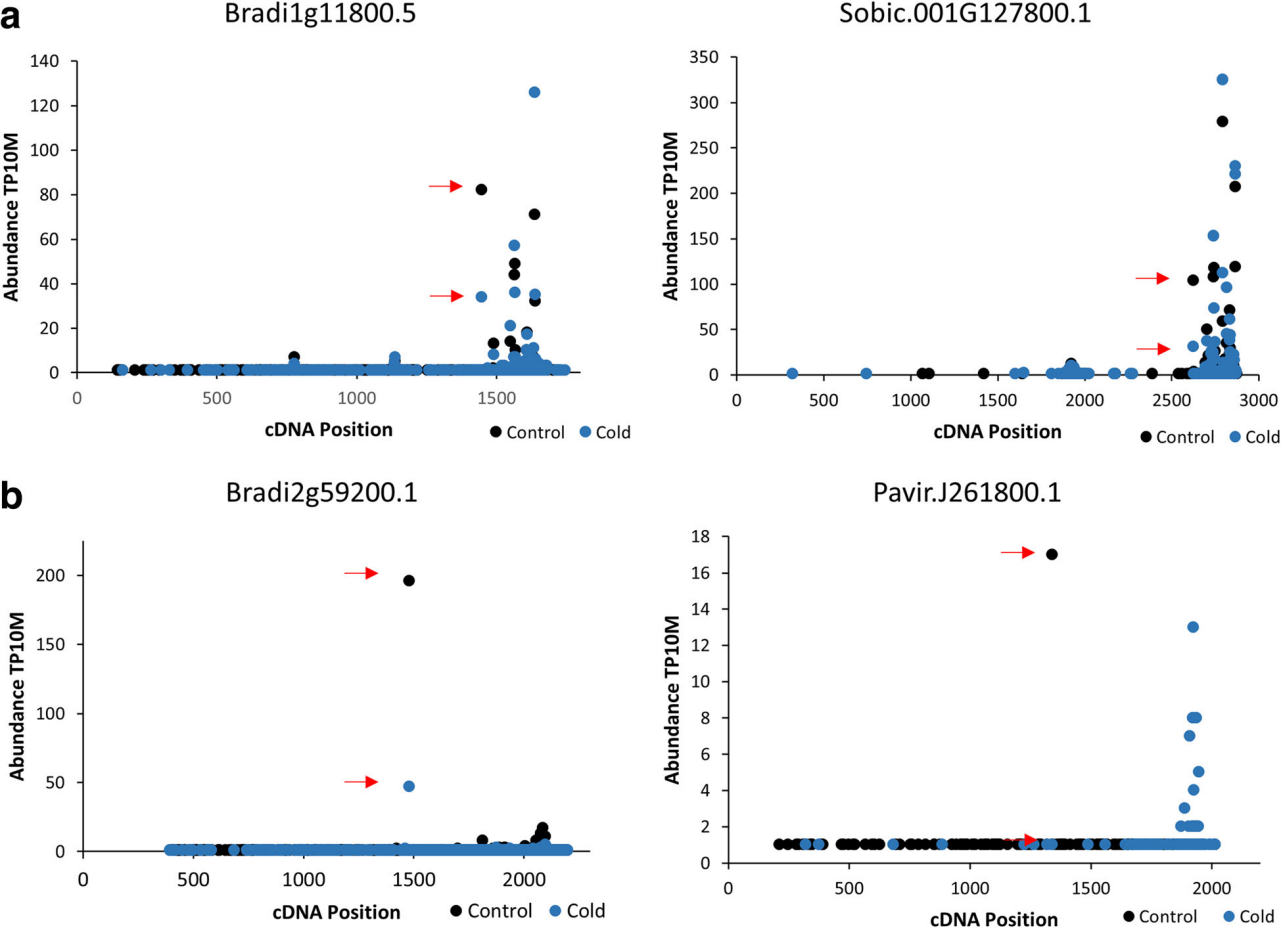
Conserved cold regulation of miRNA guided cleavages in sorghum and switchgrass. PARE data of a sorghum homologue of Bradi1g11800.5 (**a**) and a switchgrass homologue of Bradi2g59200.1 (**b**) suggest the regulation of these cold regulated miRNA guided cleavages may be conserved in these biofuel crops

MiR169a showed a steady decrease in expression after 2-3 h of cold stress in both biological replicates, which correlates well with the increased abundance of its target, *NF-YA*. Additionally, this target was found to be conserved in sorghum and PARE data from cold treated plants suggesting the cold responsiveness may be conserved as well. The changes in abundance of the PARE sequence mapping to the predicted target site in sorghum PARE libraries mirrored the changes observed in Brachypodium (Fig. 9a).

MiR394ab exhibited an increase in expression under cold conditions starting at the 2 h time point, the opposite result one might expect of a canonical miRNA/target relationship. This type of regulation is known as an incoherent negative feed-forward loop, where under a certain condition the expression of both the miRNA and its target mRNA are induced. In these circumstances, the miRNA is used to modulate the expression of the target mRNA [38]. This relationship has been observed under cold stress conditions in Arabidopsis for Ath-miR394 and its target *LCR*, a homologue of Bradi2g59200.1 [39]. Furthermore, PARE data give evidence for this regulatory mechanism being conserved in switchgrass with the abundance of the PARE sequence mapping to the predicted target site decreasing in abundance under cold conditions (Fig. 9b).

## Discussion

Through this study we have come to a deeper understanding of the miRNAs of these grasses in multiple ways. Small RNA libraries from switchgrass allowed for the identification of 28 conserved miRNAs. The deep sequencing of PARE libraries from Brachypodium yielded experimental evidence for the miRNA guided cleavage of over 9000 additional predicted target sites as compared to our previous study. Analysis of PARE library data from sorghum and switchgrass revealed that evidence for cleavage of as many as 1000 of those Brachypodium targets was found in these bioenergy crops. To our knowledge, this study represents the first published degradome data for sorghum and switchgrass, as well as the first study of conserved miRNA targets among these species. Combining PARE and RNA-seq data allowed us to identify 44 unique Brachypodium miRNA targets which exhibited changes in PARE which could not be explained by changes in transcription, and gives evidence for miRNA involvement in multiple stress response pathways. Cold stress time course experiments allowed us to characterize the abundances of miR169a, miR394ab, and their targets throughout the first 36 h of the stress response. Prior to this, experimental evidence for the involvement of miR169a and miR394ab in the cold stress response in Brachypodium was lacking.

### Identification of Brachypodium miRNA targets via PARE

The number of predicted Brachypodium targets with PARE evidence for cleavage increased drastically compared to our previous study. We believe this is due to a few main factors. Firstly, not only have the number of annotated transcripts in Brachypodium increased but so has the number of annotated miRNAs. Going from 116 to over 500 miRNAs certainly increased the number of predicted miRNA targets. Furthermore, the use of not only 14 additional PARE libraries, but also Illumina HiSeq sequencing technology instead of GAII yielded over 2 billion PARE reads for analysis. This drastic increase in sequencing depth allowed us to capture evidence for cleavage of a much larger number of Level 1 targets. We initially questioned whether this increase in library depth warranted altering the prominence criteria. We found that while increasing the minimum required abundance to 2 TP10M instead of 1 TP10M did reduce the number of Level 1 target sites to 1180, this also caused the loss of a key known miRNA/target interaction, miR168 targeting AGO1 (Additional file 2: Figure S3). Knowing this, we felt it was best to leave the previously established prominence criteria in place. Although we would not claim that all level 1 targets are the result of a miRNA-guided cleavage, presenting all the data allows other researchers to decide which they want to pursue. The number of Level 2 and Level 3 targets also increased by multiple fold and 18 new Level 4 were identified. Experimental evidence for this many miRNA targets greatly improves our knowledge of how these small RNA molecules affect post-transcriptional gene regulation in Brachypodium.

### Conservation of miRNA targets in sorghum and switchgrass

To our knowledge this study represents not only the first genome-wide analysis of miRNA targets and degradome sequencing in sorghum and switchgrass, but also the first genomic analysis of conservation of miRNA targets with evidence for cleavage in plants. The large number of conserved targets identified across all three grasses gives credence to the use of Brachypodium as a model to study miRNA/target interactions for these bioenergy crops as well as other monocots. Interestingly, while switchgrass only had 28 miRNAs used for target prediction as compared to 152 for sorghum, switchgrass exhibited a greater number of Level 3 and 4 conserved targets. This becomes less surprising when you consider the vast majority of targets in those categories for all three species were predicted to be targeted by highly conserved miRNAs such as miR156, miR169, and miR172 (Additional file 1: Tables S2 S4 and S5). Also, the switchgrass miRNAs presented here were filtered to meet the more rigorous criteria recommended very recently for plant miRNA annotation [36]. This high level of conservation observed in miRNAs, miRNA targets, as well as the prominence of the miRNA guided cleavage, not only supports the use of Brachypodium as a model but also suggests the stress regulated mechanisms are likely to be conserved. XRN4 mutants could increase the sensitivity of these analyses as it did for Arabidopsis [6], however, we are not aware of any viable XRN4 monocot mutant.

### Stress responsive miRNAs and targets

A previous study using the ABR5 ecotype of Brachypodium showed an increase in miR393ab under cold conditions, a decrease in abundance of one of the TIR1-like transcripts miR393ab was predicted to target (Bradi5g08680.1), and no change in the abundance of the other TIR1-like predicted target (Bradi2g35720.1) [19]. However, in the Bd21 ecotype used in this study, a decrease in miRNA guided cleavage at the predicted target site for both transcripts in both biological replicates was observed, and RNA-seq analysis indicated an increase in the abundance of Bradi2g35720.1 but no change in Bradi5g08680.1. Additionally, we did not observe a significant change in miR393ab abundance during the time course. These differing results are of particular interest due to the increased cold tolerance exhibited by the ABR5 ecotype compared to the Bd21 ecotype used here. Zhang et al. demonstrated a survival rate of ~ 80% for ABR5 plants after being exposed to -5 °C for 6 h, while the survival rate for Bd21 was less than 10% [19]. It is possible that the differences observed in the regulation of miR393ab and its targets between these two ecotypes contribute to the difference in cold tolerance, but a deeper investigation is needed to say for certain. Not only does the involvement of miR393 and its targets in the stress responses extend to other plants such as rice, Arabidopsis, Medicago (*Medicago truncatula*), and common bean (*Phaseolus vulgaris*), but the overexpression of a miR393-resistant form of TIR1 was shown to enhance salt tolerance in Arabidopsis [40–46].

The miR169 family is both highly conserved among plants, and consists of a large number of variants. In Brachypodium, 16 unique mature miR169 sequences arise from 14 precursors. While this is the first time that the miR169a variant has been reported to be stress responsive in Brachypodium, other variants have been shown to be regulated under abiotic stress conditions in Brachypodium as well as other plants [18, 22, 23, 40, 47–50]. Some differences exist in the reported direction of miR169 regulation under stress. While our results indicated a decrease in miR169a expression took place after 2-3 h of cold stress, which correlated with an increase in abundance of the NF-YA target, another study based on drought microarray data showed miR169g upregulated by stress in rice [40]. In Arabidopsis, an investigation of drought stress reported the down regulation of miR169a and miR169c [23]. Time course experiments done on maize found miR169 variants to be downregulated in the short term (48 h) but upregulated over long term (15 day) conditions of drought stress, salt stress and ABA treatment [49]. This difference in regulation over time could explain the previous discrepant results; our time course experiments extended to 36 h and the results are in line with the short term observations in maize. MiR169 targets the large family of NF-Y transcription factors and stress induced early flowering in Arabidopsis has been shown to be regulated by an increase in miR169 and repression of the AtNF-YA2 target [48]. It is possible that the early repression of miR169 is an attempt to repress flowering in case the stress is temporary; however, if the stress continues, a later induction of miR169 triggers early flowering so that the plant can complete its life cycle faster. Despite the discrepancies in the direction of miR169 regulation, under various stress conditions, in different plants, and across alternating time points, it is clear that miR169 plays a significant role in the stress responses. Overexpression of NF-Y family members has been shown to confer drought tolerance in maize as well as tolerance to drought, cold, flooding, and heat stresses in Arabidopsis [23, 47, 51, 52]. Given these results along with PARE data suggesting this regulation is also conserved in Sorghum (Fig. 8a) we would expect a similar increase of stress tolerance in a Brachypodium overexpressor.

A number of miRNAs and targets in the “Inverse” group were implicated in the drought and submergence stress responses, and would be of interest to characterize further in the future. Not only has miR396 been shown to decrease under drought conditions in rice [49], but an Arabidopsis miR396 overexpressor was shown to have lower densities of stomata and increased drought tolerance compared to wild-type. A study done in Brachypodium exposed to drought stress revealed an increase in miR390 [21], a miRNA which triggers the production of tasiRNA (TAS3-derived trans-acting small interfering RNA) which target various auxin response factors [15]. Our results give additional evidence for the association of miR390 with the drought stress response of Brachypodium. Evidence for miR396 involvement in reprogramming leaf growth under drought conditions in Brachypodium has been reported [22], but an overexpressor has not yet been generated. Increased levels of miR171 were observed in submerged maize plants [50], whereas its association with the Brachypodium submergence response is novel.

While miRNAs are often mentioned as being mostly modulators of gene expression rather than primary regulators, more examples of the latter are found in literature as larger changes are easier to validate experimentally. Our analysis was able to identify both miRNAs which act as primary regulators of mRNA targets, as well as a much larger number of miRNAs for which PARE and RNA-seq evidence suggests act to keep target mRNAs at steady state levels during the stress responses. The “Unchanged” category of miRNA targets was the largest overall group. Even though the miRNAs may not be the primary regulators of the target mRNAs, the importance of these interactions should not be discounted, especially when it comes to the identification of gene candidates for the genetic engineering of more stress tolerant plants. While our analysis put miR394ab and its target, an Ath-LCR homolog in the “Inverse” category, we know that the miR394ab is not the primary regulator of this target based on research done in Arabidopsis [39] and the nature of these incoherent interactions [38]. Despite this, it was found that both an Ath-miR394a overexpressor as well as an Ath-LCR loss of function mutant exhibited greater tolerance to cold stress as compared to wild type [39]. MiRNAs and target mRNAs in the “Unchanged” category are prime candidates for similar research, and there is much potential to expand this type of analysis to other plants and identify a greater number of these fine tuning miRNA/target interactions. The majority of miRNAs targeting transcripts in the “Inverse” and “Unchanged” categories are members of highly conserved miRNA families (Fig. 6a) and the targets represent mostly transcription factor families, such as the NF-Ys, SPLs, GRFs, and ARFs, that are also highly conserved among plants. After observing this high level of conservation of miRNAs and targets, the evidence for involvement in multiple stress responses, and the identification of candidates that have already been shown to confer stress tolerance in transgenic plants, it is clear that these data will guide future researchers attempting to yield similar results.

## Conclusions

The knowledge gained from the identification of conserved and PARE validated miRNA targets in Brachypodium, sorghum, and switchgrass deepens our understanding of the miRNA regulatory pathways in these plants as well as how those pathways are conserved. These results will encourage future investigations of miRNA targets in these plants as well as other monocots. The use of PARE to find instances of stress regulated miRNA guided cleavage gives us a much more complete view of the complex regulatory networks of the stress responses. The ability to identify changes in miRNA guided cleavage even when the miRNA is not the primary regulator of the target mRNA abundance vastly increases the number of potential targets for the development of stress tolerant transgenic crops.

## Methods

### Plant growth and stress treatments

Brachypodium Bd21 seeds were germinated in soil and the plants were grown in a growth chamber at a constant 20 °C, under a 20 h light 350 μE·m − 2·s − 1, 4 h dark cycle. On day 21, 10 h after light started, plants were subjected to various stress treatments. For heat stress, plants were placed at 40 °C. For drought stress, plants were removed from soil and the roots were dried with paper towels. For submergence stress, plants were submerged completely with at least 5 cm of tap water covering the top of the plants. For cold stress, plants were placed at 4 °C. For stress libraries, above ground tissue was sampled after 12 h of stress; for the cold stress time courses, samples were taken at 0, 1, 2, 3, 6, 12, 24, and 36 h. Switchgrass (AP13) was grown under greenhouse conditions with an average day temperature of 26 °C and an average night temperature of 20 °C. Stress treatments were carried out two months after propagation from cuttings and the second and third leaf blades from the top were sampled. After germination, *Sorghum bicolor* (BTx623) seedlings were transferred to soil in a growth chamber. After 14 days of growth (12 h light at 28 °C, 12 h dark at 25 °C), cold treatments were carried out and the above-ground tissue was harvested. Switchgrass and sorghum cold treatments at 4 °C were performed for 24 h. For switchgrass drought experiments, the drought sample was harvested 11 days after watering was stopped. Plants subjected to recovery were then watered, and on the 12th day they were sampled.

### Library construction

PARE and smRNA libraries were constructed as previously described [6] except the Illumina HiSeq 2000 sequencing platform was used. RNA-seq libraries were constructed using the ScriptSeq RNA-Seq Library Preparation Kit from Illumina (Cat#SSV21124) and sequenced using Illumina HiSeq 2500.

### Reference genomes

For bioinformatic analyses and library mapping the *Brachypodium distachyon* v3.1, *Sorghum bicolor* v3.1.1, and *Panicum virgatum* v4.1 genomes and annotations from DOE-JGI [26–28] were used.

### smRNA library analysis and miRNA discovery

Sequencing data was trimmed by removing adapter sequences, and mapped to the *Panicum virgatum* genome using Bowtie [24]. Our miRNA discovery pipeline [6] with updated criteria [36] was used to identify conserved miRNAs in switchgrass. Briefly, small RNA sequences 20 to 24 nucleotides with an abundance of ≥10 TP10M, and ≤ 20 total genome hits, were evaluated for potential miRNA and miR* pairing using modified miREAP [53] only if they matched known mature miRNAs from miRBase 21. The total abundance of reads matching the sense strand was divided by the total abundance of all reads matching both strands to calculate strand bias. The sum of the abundances of the top two most abundant reads was divided by the total abundance of all matching reads to calculate abundance bias. Only those precursor sequences with a strand bias of ≥0.9 and an abundance bias of ≥0.7 were selected for stem-loop structure prediction via UNAFold [54]. Precursor sequences less than 300 nt in length, with ≤5 mismatch positions, only three or fewer of which were nucleotides in asymmetric bulges, in the miRNA:miRNA* pairing were considered conserved miRNAs for subsequent target prediction.

### PARE and RNA-seq library analysis

PARE libraries were analyzed as previously described [55] except perfect matches were required when using Bowtie [24] to map reads to the genome. RNA-seq libraries were mapped to the Brachypodium genome using STAR [29] with ENCODE RNA-seq standard parameters. Differential expression was calculated using RSEM and edgeR [56, 57].

### miRNA target prediction and characterization

Two programs were used for miRNA target prediction. The 2011 version of psRNATarget [34] with the following parameters was used: maximum expectation was set to 5.0, length for complementarity scoring (hspsize) was set to the length of the miRNA, and the range of central pairing was set at 10 to 11. Targetfinder was used with a prediction score cutoff value of 4 [58]. Primary cDNA transcripts from *Brachypodium distachyon* along with mature Bdi-miRNA sequences from miRBase 21 [59] were used as input for both psRNATarget and Targetfinder. Homologs of Brachypodium target mRNAs with PARE evidence for cleavage were identified in sorghum and switchgrass using BLAST [60] and cDNA sequences were used as inputs for the target prediction programs. Mature Sbi-miRNA sequences from miRbase 21 were used for sorghum and the conserved miRNAs we identified, as described previously, were used for prediction in switchgrass. Predicted miRNA target sites were then characterized based on the criteria previously described and outlined in Fig. 1 [6].

### Identification and characterization of stress regulated miRNA guided cleavage events

miRNA guided cleavage events undergoing regulation during the stress responses were identified using the pipeline outlined in Fig. 4. These were then characterized into the groups described in Fig. 5 based on the differential expression of target mRNAs observed in RNA-seq data as calculated by edgeR [57].

### qRT-PCR and splint ligation mediated miRNA detection

qRT-PCR reactions were performed in triplicate using SYBR Premix Ex Taq II from Takara (CAT#RR820L). Relative expression was calculated using the ∆∆Ct method with UBC18 for normalization. Splint-ligation mediated miRNA detection was performed using the USB®miRtect-IT™ miRNA Labeling & Detection Kit (CAT#76400) with miR168 used as a loading control.

## Additional files

Additional file 1: **Table S1.** RNAseq Libraries. Summary statistics of Brachypodium RNAseq libraries. **Table S2.** BDI Prominence Lvls. miRNA target prediction scores and prominence data for predicted Brachypodium miRNA targets. **Table S3.** SWI miRNAs. Genomic location, folding data, and sequences of conserved miRNAs found in switchgrass**. Table S4.** SWI Prominence Lvls. miRNA target prediction scores and prominence data for predicted switchgrass miRNA targets. **Table S5.** SBI Prominence Lvls. miRNA target prediction scores and prominence data for predicted sorghum miRNA targets**. Table S6.** Inverse and Unchanged. miRNA target prediction scores, annotation information, PARE data, and RNAseq data for all miRNAs/mRNA-targets in the “Inverse” and “Unchanged” groups. **Table S7.** Primers and Oligos. Primers and oligos used in this study. (XLSX 1498 kb)

Additional file 2: **Figure S1.** D-Plots of the cold regulated inverse group miRNA targets. PARE data showing evidence for cold regulation of the miRNA guided cleavages of (A) Bradi2g59200.1, (B) Bradi1g11800.5, and (C) Bradi2g35720.1. An additional biological replicate of what is shown in Fig. 6. Red dots indicate the PARE sequences mapping to predicted target sites. **Figure S2.** Characterization of cold regulated miRNAs and mRNA targets in Biorep #2. Complementary to Fig. 8. **Figure S3.** Bdi-miR168 Targeting AGO1a. D-Plot of PARE data showing evidence for cleavage of the AGO1a transcript (Bradi3g51077.3) via miR168. Despite this cleavage event being highly conserved it is only Level 1 prominence. Red dot indicates the PARE sequence which mapped to the miR168 target site. (PPTX 1715 kb)

## Abbreviations

AGO: ARGONAUTE protein
D-Plot: Degradation plot
mb: Megabase
miRNA: microRNA
PARE: Parallel analysis of RNA ends
PCR: Polymerase chain reaction
RISC: RNA-induced silencing complex
TP10M: Transcripts per 10 million.
