# Supplementary material for: Analysis of Brachypodium miRNA targets: evidence for diverse control during stress and conservation in bioenergy crops
Source: BMC Genomics. 2018 Jul 20;19:547. doi: 10.1186/s12864-018-4911-7 (PMC6053804; doi:10.1186/s12864-018-4911-7)
Supplement: Supplementary file 2 — Figure S1. D-Plots of the cold regulated inverse group miRNA targets. PARE data showing evidence for cold regulation of the miRNA guided cleavages of (A) Bradi2g59200.1, (B) Bradi1g11800.5, and (C) Bradi2g35720.1. An additional biological replicate of what is shown in Fig. 6. Red dots indicate the PARE sequences mapping to predicted target sites. Figure S2. Characterization of cold regulated miRNAs and mRNA targets in Biorep #2. Complementary to Fig. 8. Figure S3. Bdi-miR168 Targeting AGO1a. D-Plot of PARE data showing evidence for cleavage of the AGO1a transcript (Bradi3g51077.3) via miR168. Despite this cleavage event being highly conserved it is only Level 1 prominence. Red dot indicates the PARE sequence which mapped to the miR168 target site. (PPTX 1715 kb) [file 12864_2018_4911_MOESM2_ESM.pptx]

## Slide 1
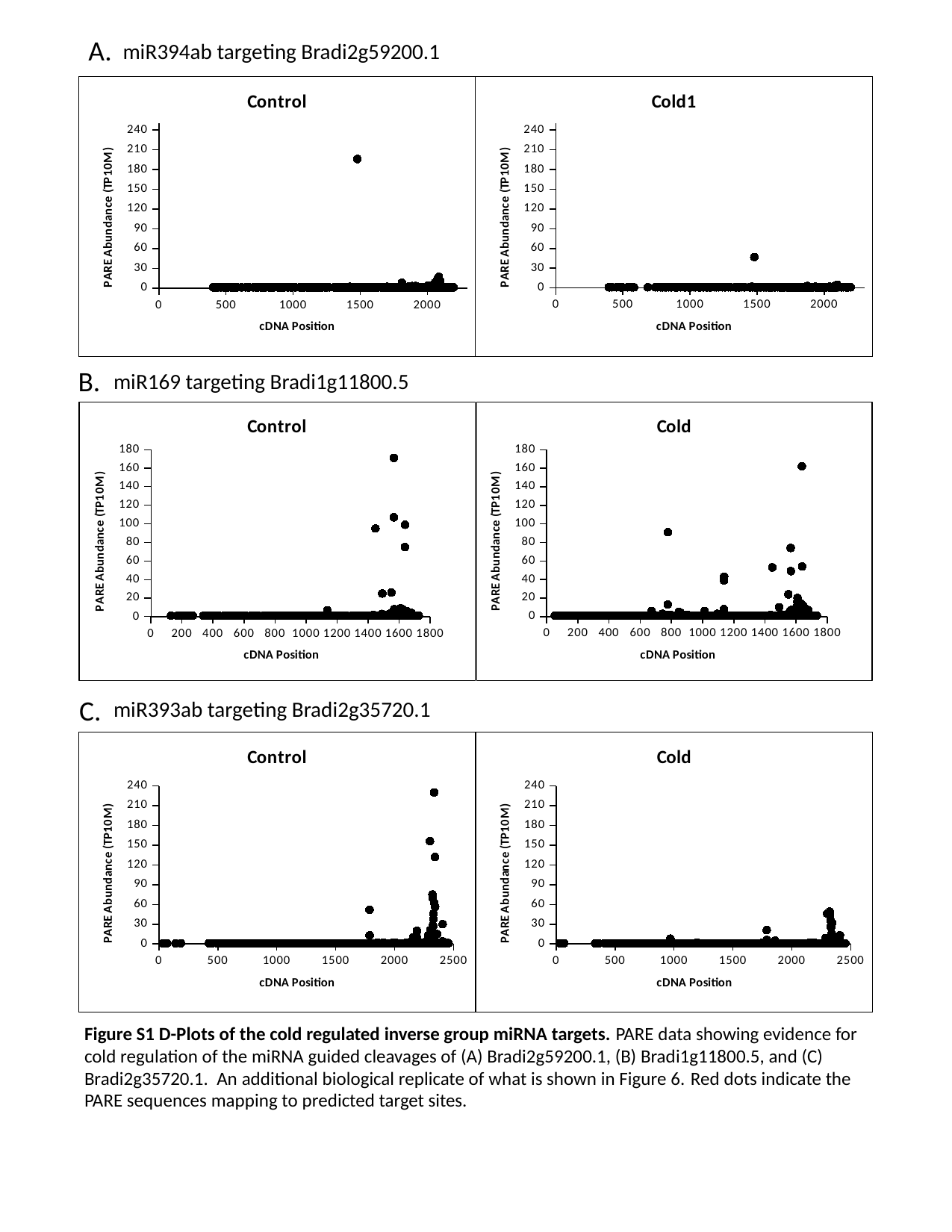

A.
miR394ab targeting Bradi2g59200.1
### Chart:
| Category | |
|---|---|
### Chart: Control
| Category | |
|---|---|B.
miR169 targeting Bradi1g11800.5
### Chart: Control
| Category | Control1 |
|---|---|
### Chart: Cold
| Category | Cold1 |
|---|---|C.
miR393ab targeting Bradi2g35720.1
### Chart: Control
| Category | |
|---|---|
### Chart: Cold
| Category | |
|---|---|Figure S1 D-Plots of the cold regulated inverse group miRNA targets. PARE data showing evidence for cold regulation of the miRNA guided cleavages of (A) Bradi2g59200.1, (B) Bradi1g11800.5, and (C) Bradi2g35720.1. An additional biological replicate of what is shown in Figure 6. Red dots indicate the PARE sequences mapping to predicted target sites.

## Slide 2
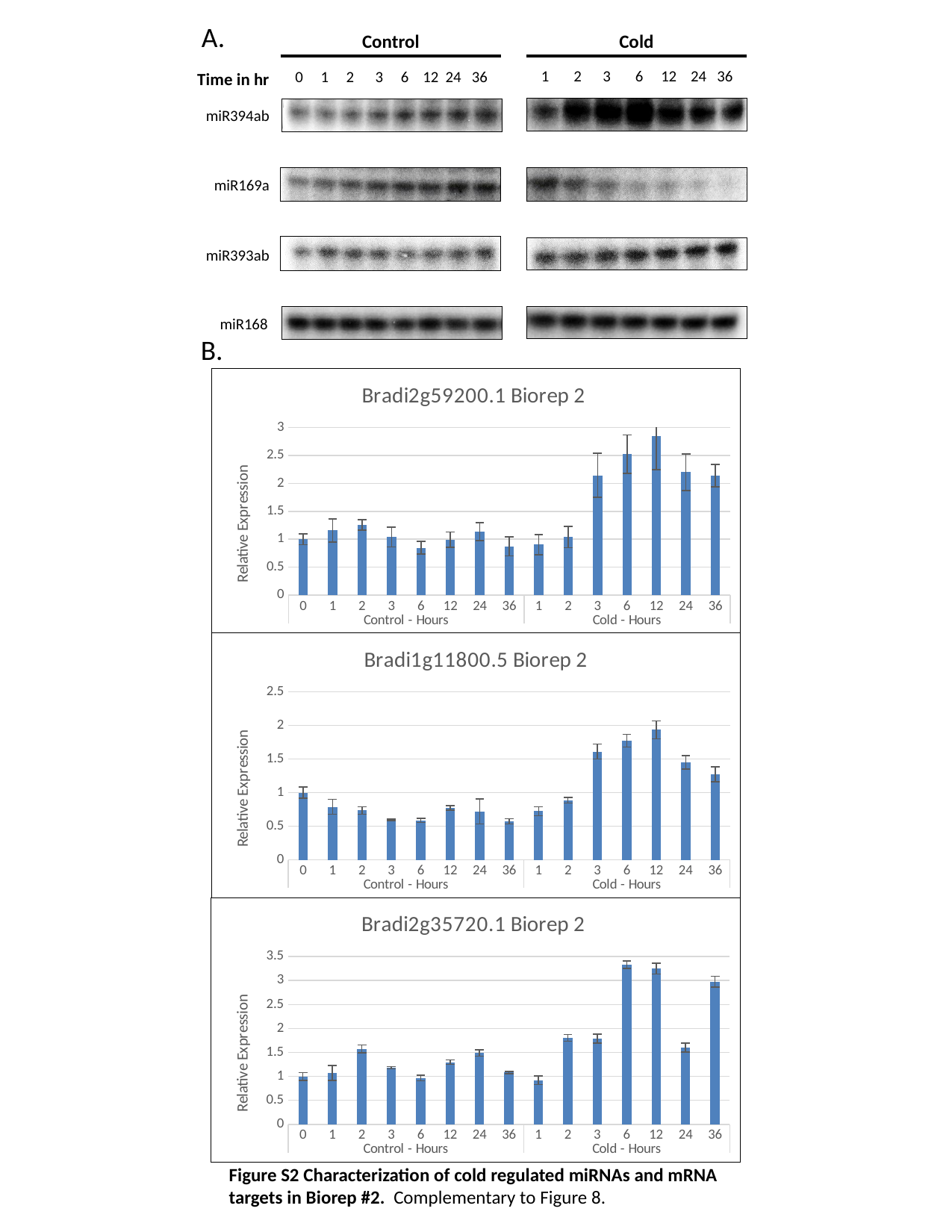

A.
Cold
Control
 1 2 3 6 12 24 36
 0 1 2 3 6 12 24 36
Time in hr
miR394ab
miR169a
miR393ab
miR168
B.
### Chart: Bradi2g59200.1 Biorep 2
| Category | |
|---|---|
| 0 | 1.0 |
| 1 | 1.15625925281442 |
| 2 | 1.25686962477325 |
| 3 | 1.03991997670579 |
| 6 | 0.84909150878516 |
| 12 | 0.994078276159445 |
| 24 | 1.13494071210368 |
| 36 | 0.871317680488249 |
| 1 | 0.904280128666036 |
| 2 | 1.0395504301073 |
| 3 | 2.14298516208167 |
| 6 | 2.52217465047517 |
| 12 | 2.84560582282421 |
| 24 | 2.19814660136206 |
| 36 | 2.13985229815776 |
### Chart: Bradi1g11800.5 Biorep 2
| Category | |
|---|---|
| 0 | 1.0 |
| 1 | 0.787812092600568 |
| 2 | 0.734130440199842 |
| 3 | 0.594367207791386 |
| 6 | 0.586685929130866 |
| 12 | 0.770838832509647 |
| 24 | 0.720230980297104 |
| 36 | 0.570841237099286 |
| 1 | 0.723341726363629 |
| 2 | 0.887590947688948 |
| 3 | 1.61210811190521 |
| 6 | 1.77501233209239 |
| 12 | 1.93482958380337 |
| 24 | 1.44980442388049 |
| 36 | 1.27301833862949 |
### Chart: Bradi2g35720.1 Biorep 2
| Category | |
|---|---|
| 0 | 1.0 |
| 1 | 1.06972785195064 |
| 2 | 1.57254607164119 |
| 3 | 1.17926485643612 |
| 6 | 0.964936465267787 |
| 12 | 1.29541985189094 |
| 24 | 1.48853170877504 |
| 36 | 1.08138527414628 |
| 1 | 0.919947728623044 |
| 2 | 1.80616770574838 |
| 3 | 1.78626043216381 |
| 6 | 3.32860808422496 |
| 12 | 3.24635689886649 |
| 24 | 1.59848121760808 |
| 36 | 2.97570059995572 |Figure S2 Characterization of cold regulated miRNAs and mRNA targets in Biorep #2. Complementary to Figure 8.

## Slide 3
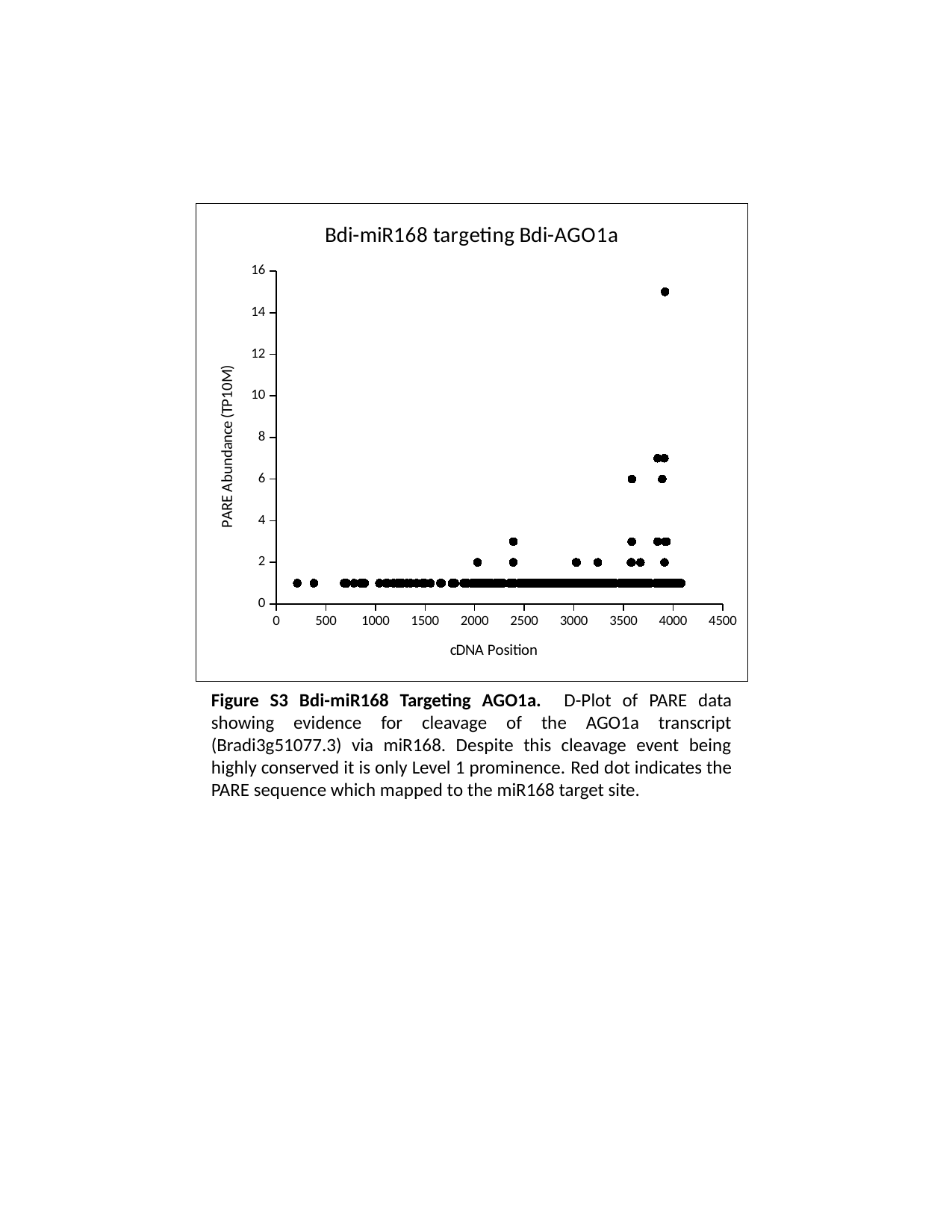

### Chart: Bdi-miR168 targeting Bdi-AGO1a
| Category | BDI507cDNA_tplot |
|---|---|Figure S3 Bdi-miR168 Targeting AGO1a. D-Plot of PARE data showing evidence for cleavage of the AGO1a transcript (Bradi3g51077.3) via miR168. Despite this cleavage event being highly conserved it is only Level 1 prominence. Red dot indicates the PARE sequence which mapped to the miR168 target site.
